# Antioxidant Therapy in Inflammatory Bowel Disease: A Systematic Review and a Meta-Analysis of Randomized Clinical Trials

**DOI:** 10.3390/ph16101374

**Published:** 2023-09-28

**Authors:** José Israel Rodrigues Junior, Joice Kelly Gomes de Vasconcelos, Lylian Ellen Militão dos Santos Xavier, Amanda da Silva Gomes, Juliana Célia de Farias Santos, Samara Bomfim Gomes Campos, Amylly Sanuelly da Paz Martins, Marília Oliveira Fonseca Goulart, Fabiana Andréa Moura

**Affiliations:** 1 Faculdade de Nutrição (FANUT), Universidade Federal de Alagoas (UFAL), Maceió 57072-970, Brazil; jose.rodrigues@fanut.ufal.br (J.I.R.J.); joice.vasconcelos@fanut.ufal.br (J.K.G.d.V.); samara.bomfim@fanut.ufal.br (S.B.G.C.); 2 Pós-Graduação em Nutrição (PPGNUT), Universidade Federal de Alagoas (UFAL), Maceió 57072-970, Brazil; lylian.militao@alicerceedu.com.br (L.E.M.d.S.X.); amanda.gomes@fanut.ufal.br (A.d.S.G.); 3 Pós-Graduação em Ciências Médicas (PPGCM/UFAL), Universidade Federal de Alagoas (UFAL), Maceió 57072-970, Brazil; juliana.santos@fanut.ufal.br; 4 Pós-Graduação da Rede Nordeste de Biotecnologia (RENORBIO), Universidade Federal de Alagoas (UFAL), Maceió 57072-970, Brazil; amylly.martins@iqb.ufal.br (A.S.d.P.M.); mofg@qui.ufal.br (M.O.F.G.); 5 Instituto de Química e Biotecnologia (IQB/UFAL), Universidade Federal de Alagoas (UFAL), Maceió 57072-970, Brazil; 6 Pós-Graduação em Ciências da Saúde (PPGCS), Universidade Federal de Alagoas (UFAL), Maceió 57072-970, Brazil

**Keywords:** ulcerative colitis, Crohn’s disease, oxidative stress, cytokines

## Abstract

The objective of this study is to assess the effectiveness of treatment for inflammatory bowel diseases in modulating oxidative stress biomarkers and cytokine levels. A systematic review of clinical trials was conducted, searching electronic databases including PubMed, Science Direct, and Scopus. After excluding articles that did not meet the inclusion criteria, 19 studies were included in the systematic review and 8 in the meta-analysis (6 for antioxidant capacity, 6 for superoxide dismutase (SOD), and 5 for lipid peroxidation analyzed through malondialdehyde (MDA) levels). SOD was significantly modulated (RR = 0.3764, 95% CI [0.0262 to 0.7267], *p* = 0.035) but not antioxidant capacity (RR = 0.3424, 95% CI [0.0334 to 0.7183], *p* = 0.0742) or MDA (RR = −0.8534, 95% CI [−1.9333 to 0.2265], *p* = 0.1214). Nonetheless, studies investigating oxidative stress biomarkers and cytokines in the context of alternative therapies for IBD treatment are still scarce. This review highlights the potential of antioxidant supplementation in IBD management and underscores the need for further investigations into its effects on oxidative stress biomarkers and cytokines to improve therapeutic approaches for IBD patients.

## 1. Introduction

Crohn’s disease (CD) and ulcerative colitis (UC), collectively known as inflammatory bowel diseases (IBD), are currently recognized as significant global public health concerns. In 2019, the Global Burden of Disease (GBD) study reported approximately 4.9 million IBD cases worldwide, with the highest prevalence rates found in China and the United States [1]. Despite the GBD study reporting an increase in the number of deaths and disability-adjusted life years (DALY), which is an index of the overall disease burden, representing the loss of one year of full health, age-standardized indicators have shown a significant reduction when compared to the prevalence identified in the 1990s. The enhancement of patients’ quality of life in IBD is primarily credited to advances in new biological therapies, specialized medical practices, and multidisciplinary treatment strategies [2].

Building upon this positive impact of multidisciplinary treatment approaches, there is a growing interest within the scientific community to identify alternative therapies that can help minimize the characteristic signs and symptoms of the disease. Among these therapies, the use of antioxidants, natural or synthetic, has gained attention due to their promising effects, particularly in animal models [3].

Oxidative stress, characterized by an imbalance between pro-oxidants and antioxidants favoring the former, plays a critical role not only in the development of IBD but also in the exacerbation of its signs and symptoms. This imbalance can result in damage to macromolecules and is graded on an intensity scale ranging from eustress (physiological stress) to distress (excessive and toxic oxidative burden) [4]. The detrimental effects of oxidative stress in IBD are manifested through a range of symptoms, including diarrhea, weight loss, ulceration, and even colorectal cancer (CRC) [5].

To assess these effects, several clinical trials have investigated substances with potential antioxidant and anti-inflammatory activity, as observed in experimental studies, in patients with IBD [6,7,8,9,10]. However, only a few studies have evaluated their impact on redox imbalance and cytokine profiles. In this context, the present systematic review with meta-analysis aims to determine the efficacy of antioxidant substances in modulating biomarkers of oxidative stress and pro- and anti-inflammatory cytokines in individuals with IBD. By summarizing the existing evidence, this study aims to offer valuable insights into the potential advantages of antioxidant treatments in IBD management and contribute to the development of targeted interventions for this complex and debilitating condition.

## 2. Methods

### 2.1. Search Strategy and Selection of Studies

The search was conducted until July 2023 in the following databases: MEDLINE (via PubMed), Science Direct, and Scopus. The following keywords were used: “inflammatory bowel disease”, “ulcerative colitis”, “colitis”, “Crohn Disease”, “antioxidant”, “Antioxidant Effects”, “Anti Oxidants”, “Agents, Antiinflammatory”, “Anti Inflammatories”, “therapy”, “treatment”, “stress oxidative,” and “redox imbalance." Boolean operators “OR” and “AND” were used adjusted according with database. All records retrieved had their titles and abstracts evaluated. Then, we evaluated titles for the removal of duplicate records. A similar search was used for the other two electronic databases. Some filters, referring to randomized trials and clinical trials and the number of humans available in each database, were used. To minimize result bias, the reference lists of relevant articles were manually searched to identify any missed publications. We included full articles that satisfied the inclusion and exclusion criteria.

### 2.2. Eligibility of Clinical Research

#### 2.2.1. Clinical Studies

Human studies with participants of both sexes, diagnosed with UC or DC, examined the effects of oral consumption of antioxidants/drugs on oxidative stress and/or cytokine markers. There was no restriction on age, the severity of the disease (mild, moderate, or severe), or the location of the intestinal lesion (proximal or distal). Studies were excluded if they evaluated pregnant or lactating women and participants with other associated comorbidities, such as diabetes and hepatic, kidney, and autoimmune diseases.

#### 2.2.2. Meta-Analysis

Randomized Clinical Trial (RCT) with participants of both sexes, aged 18 years or older, diagnosed with UC or DC, and oral consumption of antioxidants/drugs on oxidative stress and/or cytokines markers. There was no restriction on the severity of the disease (mild, moderate, or severe) or the intestinal lesion location (proximal or distal). Studies were excluded if they evaluated pregnant or lactating women and participants with other associated comorbidities, such as diabetes and hepatic, kidney, and autoimmune diseases.

This Systematic Review was registered in the International Prospective Register of Systematic Reviews (PROSPERO) nº CDR42022335357.

### 2.3. Data Extraction

#### 2.3.1. Clinical Studies

IBD clinical situation; number of randomized individuals (n)/age (years); intervention; dose and time of intervention; oxidative stress markers and cytokines effect.

#### 2.3.2. Meta-Analysis

RCTs included in the meta-analysis were required to provide data on oxidative stress or cytokine biomarkers. The mean values of the biomarkers were then normalized by their standard deviation (SD) to standardize the data and reduce discrepancies resulting from different analytical methods. For studies that presented data using the standard error of the mean (SEM), the values were recalculated to the standard deviation (SD) for uniformity in the normalization process. Meta-analyses were conducted to assess the levels of superoxide dismutase (SOD), malondialdehyde (MDA), and antioxidant capacity.

#### 2.3.3. Assessment of the Risk of Bias

The risk of bias of the randomized clinical trials (RCT) included was evaluated according to the Cochrane risk of bias tool. The risk of bias was independently assessed in six domains: random sequence generation, allocation concealment, blinding of participants and professionals, blinding of outcome assessors, incomplete outcomes (intention-to-treat or per-protocol analysis), and selective outcome reporting. All studies that did not present a registered clinical protocol were classified as high-risk of bias in the “selective outcome report” domain. For non-randomized controlled studies, the ROBINS-I tool was used in seven domains: confounding, participant selection, classification of interventions, deviations from intended interventions, missing data, measurement of outcomes, and selection of the reported result.

#### 2.3.4. Statistical Analysis

As all the metanalyzed variables were categorized, the relative risk (RR) between groups for each variable was calculated for each study. Study weights were assigned according to the inverse variance method, and calculations were based on a random-effects model. An alpha value of 0.05 was adopted.

Statistical heterogeneity among the studies was tested using the Cochran Q test, and inconsistency was assessed using I2 statistics. Whenever a result showed heterogeneity, it was explored by repeating the analysis with the removal of one study at a time to assess whether a particular study explained the heterogeneity. All analysis were conducted using the Jamovi^®^ 2.3.26 program.

## 3. Results

### 3.1. Search Results

**Figure 1:**
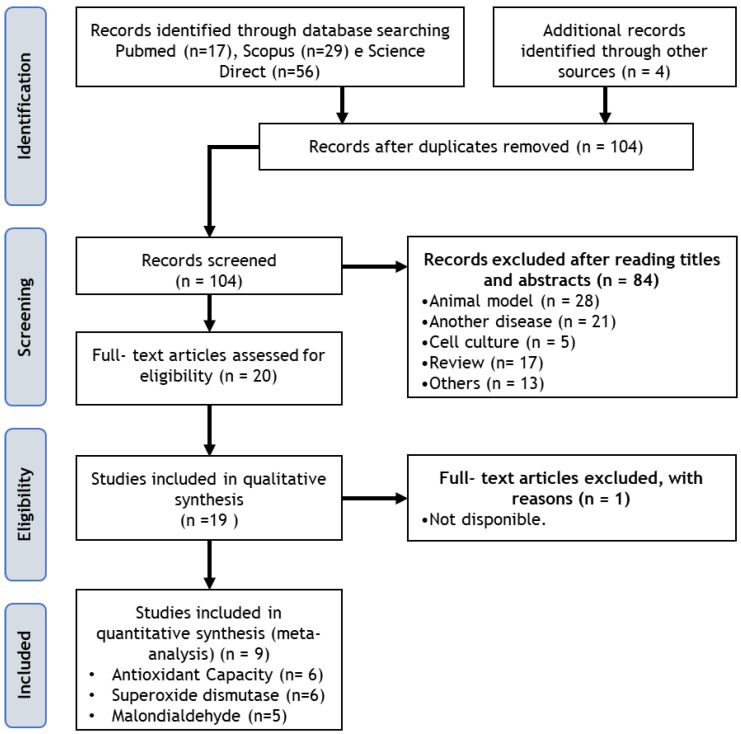
Flow diagram of study selection.

In this systematic review with meta-analysis, a total of 19 studies were identified according to the predefined inclusion criteria (Figure 1). Among them, 9 studies (47.3%) focused solely on patients with UC [11,12,13,14,15,16,17,18,19], 6 studies (31.6%) included only patients with DC [20,21,22,23,24,25], and 4 studies (21.0%) encompassed both diseases (CD and UC) [26,27,28,29]. Most of the studies (n = 17; 89.5%) involved adult patients, while 2 studies (11.5%) were specifically conducted on children and adolescents [24,25]. The selected studies exhibited diverse designs, with 15 studies (78.9%) being double-blind, placebo-controlled randomized trials [11,12,14,15,16,17,18,19,20,21,22,23,26,27,28].

A wide range of substances were tested in the included studies, including micronutrients such as antioxidant vitamin complexes [21] or isolated vitamins [13,22,29], zinc [20], the amino acid glutamine [23], functional foods such as flaxseed [14], and omega-3 fatty acids [11,17,20]. The polyphenols [12,24] or polyphenol-rich foods [16,19], plant extracts [15,26,27] and probiotics [28] were also investigated. Co-enzime Q10 and azathioprine—a traditional medication used in the treatment of IBD—were investigated by [18,25] (Table 1).

The period of treatment varied from 2 [14] to 12 weeks [16,24,25,26,27,28], with the latter being the most common intervention time among the evaluated studies.

Regarding the evaluated biomarkers of antioxidant defense, prominent ones included superoxide dismutase (SOD) [11,12,17,19,20,21,24,25,26], glutathione peroxidase (GPx) [17,19,21,24], and catalase [11,17,25], as well as antioxidant capacity [11,12,13,15,16,19,21,27]. Lipid membrane damage (lipid peroxidation—PL) was the most investigated macromolecular damage by the authors [11,12,15,16,17,19,22,23,24], while only two studies assessed transcription factors such as factor nuclear kappa B (NFκB) [15] and nuclear factor erythroid 2-related factor 2 (Nrf2) [25].

Surprisingly, the anti-inflammatory action mediated by cytokines was not extensively investigated among the studies. Only five studies explored the impact of interventions on cytokines: IL-6 [14,17,29], tumor necrosis factor alpha (TNF-α) [15,29], IL-2 [17,29], IL-1β [17,29] and IL-10 [18,29]. Notably, omega-3 supplementation (4300 mg/d for 8 weeks) reduced IL-1β levels; riboflavin (100 mg/d for 3 weeks) attenuated IL-2 levels (although it did not alter IL-6, IL-10, TNF-α, and IL-1β); and coenzyme-Q10 (200 mg/d for 8 weeks) not only reduced IL-17 levels but also increased the levels of IL-10, known for its anti-inflammatory properties.

Overall, the included studies shed light on the potential effects of various interventions on oxidative stress and inflammatory biomarkers in IBD patients. However, further research is required to fully understand the precise mechanisms and potential clinical implications of these interventions.

### 3.2. Risk of Bias

The risk of bias analysis for the included studies is presented in Table 2 and Table 3. Among the fourteen RCT studies included, ten were classified as having a low risk of bias. As for the three non-randomized controlled studies, two were classified as having a low risk of bias, while one was rated as moderate due to certain domains that might potentially influence the results analyzed in this meta-analysis.

### 3.3. Randomized Clinical Trial: Meta-Analysis

#### 3.3.1. Antioxidant Capacity

**Figure 2:**
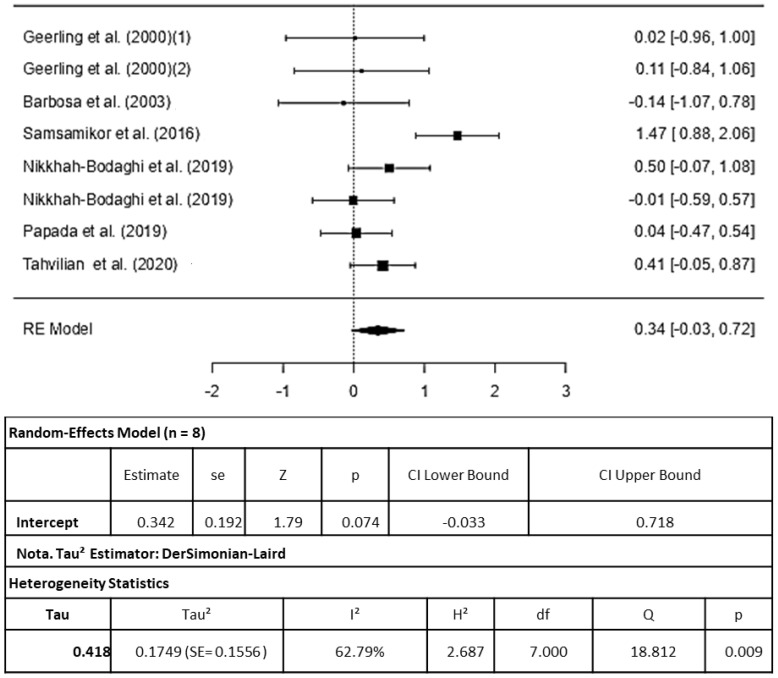
Forest plot for antioxidant capacity induced by inflammatory bowel disease therapy, according to a randomized clinical trial included in the meta-analysis Legend: df (Degrees of Freedom); H^2^ (H-squared); Q: heterogeneity test; SE: standard error; Tau^2^ (Tau squared) [11,12,15,16,19,21].

Six studies were included in the analysis (the study by [21] analyzed two intervention groups). The study by [24] was not included in the meta-analysis due to its inclusion of children and adolescents, which was a criterion for exclusion in this study. On the other hand, the RCT conducted by [13], while being an RCT, was not eligible for inclusion because it did not compare the treatment to a placebo but instead compared two different doses of Vitamin D, making it unsuitable for the treatment versus non-treatment comparison required for this analysis (Figure 2).

The observed standardized mean differences ranged from −0.1429 to 1.4691, with the majority of estimates being positive (75%). The estimated average standardized mean difference based on the random-effects model was = 0.3424 (95% CI: −0.0334 to 0.7183). Therefore, the average outcome did not differ significantly (z = 1.7857, *p* = 0.0742), indicating that there was no protective effect of the antioxidants included in this meta-analysis on the total antioxidant capacity.

According to the Q-test, the true outcomes appear to be heterogeneous (Q(7) = 18.8116, *p* = 0.009, tau^2^ = 0.1749, I^2^ = 62.7889%). A 95% prediction interval for the true outcomes is given by −0.5594 to 1.2443. Hence, although the average outcome is estimated to be positive, in some studies, the true outcome may in fact be negative. An examination of the studentized residuals revealed that one study [12] had a value larger than ± 2.7344 and may be a potential outlier in the context of this model. According to Cook’s distances, one study [12] could be considered to be overly influential. Neither the rank correlation nor the regression test indicated any funnel plot asymmetry (*p* = 0.9049 and *p* = 0.4033, respectively).

#### 3.3.2. Superoxido Dismutase

**Figure 3:**
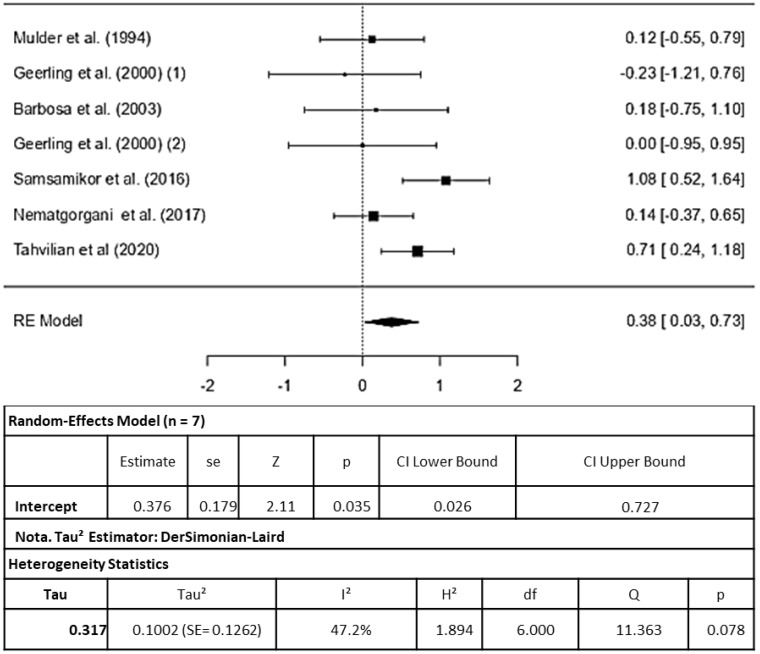
Forest plot for superoxide dismutase induced by inflammatory bowel disease therapy, according to a randomized clinical trial included in the meta-analysis. Legend: df (Degrees of Freedom); H^2^ (H-squared); Q: heterogeneity test; SE: standard error; Tau^2^ (Tau squared) [11,12,19,20,21,26].

Six studies were included in the SOD analysis (the study by [21] analyzed two intervention groups). The study by [17] despite being an RCT, was not included in the meta-analysis due to the absence of standard deviation (SD) data for SOD in its results, which rendered the normalization of the data unfeasible. Similarly, the RCT conducted by [25] could not be included because its results were presented graphically without providing mean and SD values (Figure 3).

The standardized mean differences observed varied from −0.2277 to 1.0802, and notably, most of these estimates (71%) were positive. The calculated average standardized mean difference, using the random-effects model, was RR = 0.3764 (95% CI: 0.0262 to 0.7267). Consequently, the average outcome significantly differed from zero (z = 2.1066, *p* = 0.035), affirming the protective effect of the therapies included in this meta-analysis on SOD.

The *Q*-test for heterogeneity was not significant; however, some heterogeneity may still be present in the true outcomes (Q(6) = 11.3631, *p* = 0.0778, tau^2^ = 0.1002, I^2^ = 47.1974%). The 95% prediction interval for the true outcomes ranges from −0.3360 to 1.0889. This means that although the estimated average outcome is positive, there is a possibility of negative outcomes in some studies. Examination of the studentized residuals showed that none of the studies had values exceeding ± 2.6901, indicating the absence of outliers within this model. Cook’s distance analysis revealed that none of the studies were excessively influential. Additionally, both the rank correlation and regression tests did not indicate any funnel plot asymmetry (*p* = 0.3813 and *p* = 0.0961, respectively).

#### 3.3.3. Malondialdehyde (MDA)

**Figure 4:**
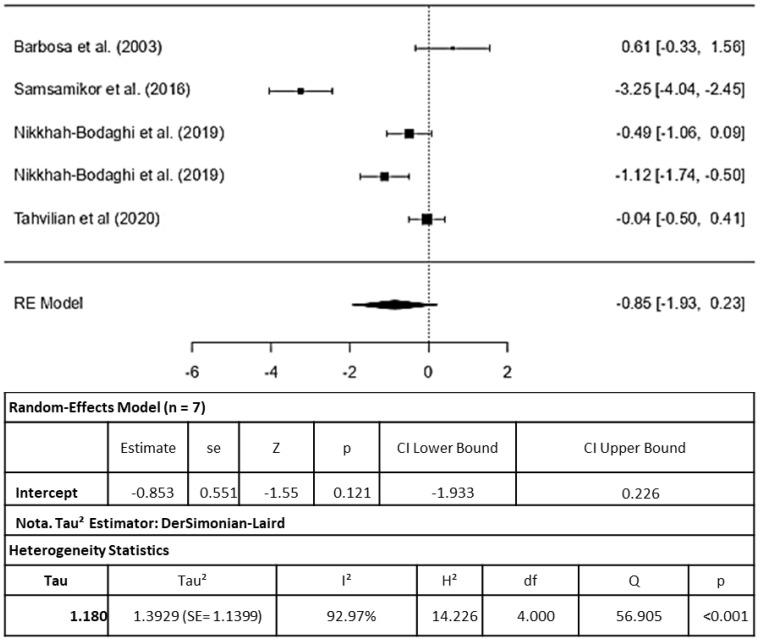
Forest plot for malondialdehyde induced by inflammatory bowel disease therapy, according to a randomized clinical trial included in the meta-analysis. Legend: df (Degrees of Freedom); H^2^ (H-squared); Q: heterogeneity test; SE: standard error; Tau^2^ (Tau squared) [11,12,15,16,19].

Five studies were included in the MDA analysis. Just like the meta-analysis for SOD, the study conducted by [17] although it was an RCT, was excluded from the meta-analysis. This was because it lacked SD data for MDA in its results, making it impossible to normalize the data for inclusion (Figure 4).

The standardized mean differences observed varied from −3.2454 to 0.6142, with a majority of these estimates (80%) being negative. The estimated average standardized mean difference, based on the random-effects model, was RR = −0.8534 (95% CI: −1.9333 to 0.2265). Consequently, the average outcome did not exhibit significant differences (z = −1.5489, *p* = 0.1214). This suggests that the use of antioxidant therapy did not significantly influence PL, as assessed through MDA levels.

Based on the Q-test, there is evidence of heterogeneity among the true outcomes (Q(4) = 56.9046, *p* < 0.0001, tau^2^ = 1.3929, I^2^ = 92.9707%). A 95% prediction interval for the true outcomes spans from −3.4062 to 1.6994. Consequently, although the average outcome is estimated to be negative, it is possible that in some studies, the true outcome may indeed be positive. An examination of the studentized residuals identified one potential outlier [12] with a value exceeding ± 2.5758 within the context of this model. According to the Cook’s distances, none of the studies appeared to exert an overly influential effect.

## 4. Discussion

### 4.1. Antioxidant Capacity

The analysis of the oxidative stress biomarkers revealed that the serum antioxidant capacity received significant attention in the included articles of the systematic review. This marker, assessed through various techniques such as total antioxidant status (TAS) [13,21,24], total antioxidant potential/capacity (TAP/TAC) [11,12,13,15,16,19,31] and total serum oxidizability (TSO) [27], holds particular importance in the context of IBD. It is deemed a primary metric for assessing the extent and capacity of oxidative stress, not just in the context of aging but also in various age-related diseases. However, according to this meta-analysis, this antioxidant marker did not undergo modulation and therefore was not influenced by the analyzed antioxidant therapy.

Serum antioxidant capacity, as an essential component of the antioxidant defense system, provides valuable insights into the overall redox balance among individuals with IBD. The human body employs a comprehensive array of mechanisms to combat redox imbalance, acting on reactive oxygen and nitrogen species and effectively repairing damage to macromolecules. This intricate defense system comprises both enzymatic and non-enzymatic endogenous components, with key enzymes such as superoxide dismutase (SOD), catalase (CAT), glutathione peroxidase (GPx), peroxyredoxin, and non-enzymatic compounds such as reduced glutathione (GSH) [31,32,33].

In addition to endogenous antioxidant defenses, the body also benefits from exogenous antioxidants obtained through dietary sources. These compounds, including α-tocopherol (vitamin E), curcumin, β-carotene, ascorbic acid (vitamin C), flavonoids, selenium, and others, are commonly found in fruits, vegetables, and grains [3,10,34]. However, when assessing the total antioxidant capacity, most methods estimate the cumulative effect of the enzymatic components of the antioxidant system, disregarding the complexity of endogenous and exogenous non-enzymatic systems.

Nevertheless, when assessing the total antioxidant capacity, it is crucial to consider the complexity of both endogenous and exogenous non-enzymatic systems. A comprehensive evaluation becomes imperative to gain accurate insights into the redox profile. In this regard, a compelling series of tests conducted by Constantini and Verhulst (2009) highlighted the significance of associating antioxidant capacity with specific markers of oxidative damage to draw reliable conclusions about the redox status across different tissues [35].

### 4.2. Superoxide Dismutase

According to the data in Table 1, it is evident that nine studies assessed the activity of SOD. Among them, six reported a significant effect of the intervention involving various antioxidants, such as pycogenol [34], resveratrol [12], *Urtica dioica* [26], omega 3 [17], saffron [19] and the drug azathioprine [25]. Notably, the study by [24] focused on children and adolescents, and [25] which analyzed genic expression, were excluded from the meta-analysis.

A noticeable increase in SOD levels/activity resulting from the use of these antioxidants in patients with IBD was observed. SOD is considered the first line of antioxidant defense and exists in three isoforms: cytosolic or copper-zinc SOD (CuZn-SOD), manganese SOD (Mn-SOD) located in mitochondria, and an extracellular form of CuZn-SOD (EC-SOD) [36]. Its role is to facilitate the conversion of the superoxide anion radical (O_2_^•−^) into hydrogen peroxide (H_2_O_2_), a less reactive oxygen species (ROS) with a longer half-life, which can diffuse through the epithelial barrier and affect neighboring cells [24].

Furthermore, it is noteworthy that H_2_O_2_, if not converted into water by the antioxidant enzymes catalase (CAT) and glutathione peroxidase (GPx), and when transition metals such as Fe^2+^ are presented, it can swiftly be converted into the extremely reactive hydroxyl radical (HO^•^) through the Fenton and Haber-Weiss reactions. HO^•^ exhibits high reactivity and causes severe damage to macromolecules, including lipid peroxidation and the breakdown of peptide bonds in intercellular junctions, leading to alterations in membrane architecture and fluidity, respectively [37]. As such, it intensifies damage to the epithelial layer and intestinal permeability loss.

This complex interplay highlights the critical role of elevated SOD levels for individuals with IBD, as they often experience compromised cellular barrier integrity and subsequent increased intestinal permeability, allowing luminal antigens, such as pathogenic bacteria and their products, particularly lipopolysaccharide (LPS), to invade the previously sterile lamina propria and submucosa [38,39,40]. Consequently, the immune response is activated, mediated by cells of innate immunity (neutrophils, macrophages, and natural killer cells) and acquired immunity (Th1, Th2, and Th17 lymphocytes), leading to the production of pro-inflammatory cytokines and reactive species, with emphasis on O_2_^•−^ synthesized by the NADPH-oxidase enzymatic complex, which is activated in the presence of neutrophils [39,41,42].

### 4.3. Malondialdehyde

According to this systematic review, eight studies analyzed lipid peroxidation through isoprostane levels [22,24], and MDA [11,12,15,16,19,23], enabling a meta-analysis of five RCTs that measured MDA levels. However, the study by [23] was excluded due to the inclusion of pediatric participants. The results of the meta-analysis demonstrated that there was no significant modulation of MDA levels by antioxidant therapy compared to the placebo group.

As previously discussed, IBD is characterized by a pronounced infiltration of immune cells into the intestinal tissue, leading to an excessive production of pro-inflammatory molecules and RONS. The primary objective of this immune response is to control microbial activity. Nevertheless, when this response becomes dysregulated, it leads to chronic activation of cellular mediators and transcription factors, such as NFκB, perpetuating the chronic oxidative and inflammatory response, resulting in severe cellular damage, including protein carbonylation, p53 mutation (p53M), DNA damage, and lipid peroxidation (LP) [43]. LP, one of the most common forms of cellular damage, is particularly generated by nitrogen dioxide radicals (^•^NO_2_), H_2_O_2_, ^•^OH, peroxynitrite (ONOO-), and hypochlorous acid (HOCl), which act on polyunsaturated fatty acids (PUFAs) and cholesterol, constituents of the colonic membrane. This process produces lipid-derived products such as 4-hydroxynonenal (4-HNE), trans-2,4-decadienal (tt-DDE), and epoxyketooctadecenoic acid, as well as the widely studied malondialdehyde (MDA) [3].

A recent review conducted by Lei et al. in 2021 reported elevated MDA levels in plasma/serum/tissues of individuals with IBD or UC, confirming the close relationship of this marker with the oxidative/inflammatory damage characteristic of these conditions [44]. However, the cause-and-effect relationship between LP and these events is not yet fully elucidated, requiring further research efforts from the scientific community [45].

LP induces cell disruption and is associated with various symptoms of IBD, including diarrhea, ulceration, necrosis, blood loss, anemia, and reduced nutrient and water absorption, resulting in weight loss and dehydration [5]. Therefore, identifying substances that can reduce this process is crucial in determining the effectiveness of an antioxidant compound.

### 4.4. Oxidative Stress and Inflammation Mediated by Cytokines

Unfortunately, only four studies [14,15,29,30] among those included in this systematic review analyzed cytokine levels, precluding the possibility of conducting a meta-analysis. The studies by [14,29] reported significant reductions in Interleukin-6 (IL-6) and Interferon gamma (IFN-γ) levels following supplementation with grounded flaxseed and riboflavin, respectively. Additionally, [18] found that coenzyme Q10 supplementation significantly altered IL-10 and IL-17 levels, differing from [25], who did observe a decrease in Il-17 levels but not in Il-10 in subjects that received selenium for 10 weeks.

The connection between oxidative stress and alterations in pro- and anti-inflammatory cytokines in IBD is well established. RONS function as signaling molecules, recruiting and stimulating effector T lymphocyte differentiation and activating pathways of pro-inflammatory mediators and cytokines (e.g., tumor necrosis factor [TNF-α] IL-1β, IL-6, IL-8, IL-17, IL-23, and IFN-γ), which have been extensively studied for their crucial role in regulating intestinal inflammation, modulating the immune response, recruiting inflammatory cells, and maintaining the chronic inflammation observed in IBD [41]. They also contribute to the expression of adhesion molecules such as intercellular adhesion molecule 1 (ICAM-1) and P-selectin [41,46].

In the context of IBD, the nuclear factor kappa B (NFκB) and active protein 1 (AP-1)/mitogen-activated protein kinase (MAPK) signaling pathways play crucial roles. Present in immune and intestinal epithelial cells, these transcription factors are essential for host homeostasis, immune tolerance, infection control, and tissue repair by inducing the expression of pro-inflammatory genes, including IL-1β, IL-6, IL-12, IL-23, nitric oxide synthase inducible (iNOS), cyclooxigenase-2 (COX-2), and TNF-α. However, their dysregulated or excessive activation can contribute to the observed chronic inflammatory response in IBD [42].

Both NFκB and AP-1/MAPK are regulated by growth factors, cytokines, RONS, and pattern recognition receptors (PRRs), especially Toll-like receptor 4 (TLR4), which, by stimulation, mainly through binding with lipopolysaccharides (LPS) from gram-negative bacteria, leads to the recruitment of innate and adaptive immune cells (macrophages, lymphocytes, and neutrophils). Subsequently, additional quantities of pro-inflammatory cytokines, RONS, chemotactic molecules (e.g., Monocyte Chemoattractant Protein-1—MCP-1), adhesion molecules (Intercellular adhesion molecule 1—ICAM-1—and vascular cell adhesion molecule 1—VCAM-1), and other inflammatory mediators (e.g., eicosanoids, platelet-activating factor, and matrix metalloproteinases) are generated, while anti-inflammatory genes such as IL-10 and transforming growth factor beta 1 (TGF-β1) are downregulated [47,48].

Pro-inflammatory cytokines such as TNF-α, IL-1, and IL-6 are known to play a role in initiating and intensifying the inflammatory response in IBD [49]. TNF-α, produced by various immune cells, is involved in the activation and recruitment of inflammatory cells to the intestine, including neutrophils and T lymphocytes [50,51]. This leads to a chronic inflammatory response in the gastrointestinal tract, resulting in intestinal tissue destruction, ulcers, fistulas, and strictures. Additionally, TNF-α disrupts the intestinal barrier by breaking the integrity of intercellular junctions, allowing antigens and bacteria from the gut lumen to enter the submucosal tissue [52]. This exacerbates the inflammatory response and contributes to its perpetuation. Furthermore, TNF-α stimulates the production of other pro-inflammatory cytokines, such as IL-1 and IL-6, creating a positive inflammatory feedback loop that amplifies the immune response and inflammation [46]. Conversely, anti-inflammatory cytokines such as IL-10 and IL-22 play a role in negatively regulating inflammation and maintaining intestinal barrier integrity [47].

The role of cytokines in IBD is so significant that various therapies aimed at their inhibition have been investigated. TNF-α inhibitors such as infliximab, adalimumab, and ustekinumab have been widely used in the treatment of CD and UC, demonstrating efficacy in inducing and maintaining clinical remission [48,53]. Other therapeutic approaches targeting cytokines, such as interleukin-12/23 and interleukin-23 blockers, have also shown significant clinical benefits in IBD patients [54,55].

On the other side, the prolonged use of these drugs resulted in side effects that limited their effectiveness and adherence, ranging from mild symptoms such as nausea and vomiting to severe conditions such as insulin resistance and hepatic toxicity. In addition to their high cost, some IBD patients become refractory to treatment, increasing the risk of complications, such as fistulas, strictures, and abscesses, especially in CD, and requiring surgical interventions, thus affecting morbidity and mortality [56,57].

This systematic review and meta-analysis have several limitations that warrant acknowledgment.

Firstly, the analyses encompassed studies involving patients with both CD and UC, including individuals in different disease phases, such as remission and active phases. The clinical heterogeneity among these studies, in terms of the types of patients included, may introduce variability into the results. It is essential to recognize that the variation in the clinical characteristics of this study populations might have influenced the overall findings.

Secondly, the assessment of oxidative stress markers and cytokines involved diverse methodologies across the included studies. These methodological variations may have introduced inconsistencies and potential biases into the interpretation of the data. However, to mitigate this issue, we applied data normalization techniques by calculating the mean and SD for each parameter, allowing for a more reliable comparison across the studies. Despite these normalization efforts, the inherent variability associated with different measurement techniques and laboratory practices remains a limitation in this analysis.

Lastly, while every effort was made to provide a comprehensive overview of the impact of antioxidants on IBD, the inclusion of only RCTs may have introduced selection bias. Excluding other study designs, such as observational studies, might limit the generalizability of the findings.

The authors are encouraged to engage in a comprehensive discussion of the results, providing insights into their interpretation concerning previous studies and the underlying hypotheses. The implications of the findings should be explored within a broader context. Additionally, it is advisable to consider potential avenues for future research in the discussion.

## 5. Conclusions

Few RCTs currently include biomarkers of oxidative stress and cytokines in the analysis of the effectiveness of potential therapies, whether traditional or non-traditional, for the treatment of IBD. Among the markers of redox imbalance that show significant modulation are antioxidant capacity and SOD; however, not MDa, a marker of lipid membrane damage.

The manipulation of ROS and cytokines represents a promising approach to managing IBD and improving the quality of life for patients. It is crucial for studies evaluating new therapeutic interventions to incorporate analyses of oxidative stress and cytokines into their assessments of therapeutic effectiveness. This integration will provide valuable insights into the potential benefits of novel treatments for IBD and contribute to the advancement of evidence-based medical interventions for this challenging condition.

## Figures and Tables

**Table 1:** Therapy for inflammatory bowel disease and its effects on biomarkers of oxidative stress and levels of pro- and anti-inflammatory cytokines.

Authors, Year	IBD	Study	Intervention	Dose and Time of Intervention	Group Subjects (n) and Age [Mean ± SD/SEM or Median (IQ)]	Oxidative Stress Markers					Cytokines	General Effects
						SOD	GPX	AOC	LP	Others		
Mulder et al. (1994) [20]	Inactive to moderately activeCD	Randomized, double blind, placebo control	Zinc aspartate	300 mg For 4 weeks	Placebo: n = 22; age = 38 y (23–55) Intervention: n = 14; age = 42 y (22–47)	NS						No changes were found in the plasma and erythrocyte Metallothionein
Geerling et al. (2000) [21]	Remission CD	Randomized, double blind, placebo control	Intervention 1 (I1): Antioxidants (AO) complex intervention 2 (I2): AO complex + omega 3 (n-3)	For 12 weeks	Placebo: n = 8; age = 38 y (30–61) I1: n = 8; age = 43 y (33–52) I2: n = 9; age = 41 y (31–56)	I1 (↑) I2 (↑)	I2(↓)	NS				AO + n-3 – decreased the proportion of arachidonic acid, and increased the proportion of eicosapentanoic acid and docosahexanoic acid in both plasma phospholipids and adipose tissue
Aghdassi et al. (2003) [22]	Remission CD	Randomized double blind, placebo control	Vit C + Vit E	Vit C: 1000 mg/d + Vit E: 800 UI/d For 4 weeks	Placebo: n = 29; age = 36.5 y ± 1.7 61) Intervention: n = 28; age = 38.3 y ± 2.9				↓			Did not alter disease activity
Barbosa et al. (2003) [11]	Mild or moderate active UC	Randomized, cross-over, placebo control	Ômega 3	4.5 g/d (90 mg of EPA + 60 mg of DHA) For 8 weeks	Placebo: n = 9; age = not informed Intervention: n = 9; age = 40 y ± 11	NS		↑	NS	Catalase: NS		Did not alter laboratory indicator or sigmoidoscopy or histology scores;
Ballini et al. (2019) [28]	DC or UC	Randomized, double blind, placebo control	Hyperbiotics Pro-15 Probiotics	12 weeks	Placebo: n = 20; age = 30–60 y Intervention: n = 20; age = 30–60 y					D-rom: ↓		↑ antioxidant defense
Akobeng et al. (2006) [23]	Active CD	Randomized, double blind, placebo control	Glutamine enriched polymeric diet	Placebo: Polymeric diet;Treatment: glutamine-enriched polymeric diet (42% of amino acid composition) For 4 weeks	Placebo: n = 8; age = 10.5 y ± 2.7 Intervention: n = 7; age = 12.2 y ± 2.8				NS			Did not alter plasma antioxidant concentrations
Kolacek et al. (2013) [24]	Remission CD	Pilot	Pycnogenol	2 mg/d For 12 weeks	Healthy control: n = 15; age = 13.9 y ± 2.0 CD patients: n = 14; age = 16.3 y ± 1.5	NS	NS		NS			Serum AOC negatively correlated with disease activity and with CRP and fecal calprotectin
Samsamikor et al. (2016) [12]	Active mild to moderate UC	Randomized double blind, placebo control	Resveratrol	500 mg/d For 6 weeks	Placebo: n = 28; age = 38.8 ± 11.6 Intervention: n = 28; age = 37.4 y ± 16.5	↑		↑	↓			↓ severity of disease activity and ↑ the quality of life
Nematgorgani et al. (2017) [26]	Mild or moderate DC and UC	Randomized double blind, placebo control	*Urtica dioica* leaf extract	400 mg For 12 weeks	Placebo: n = 29; age = 38.3 y ± 13.3 Intervention: n = 30; age = 36.6 y ± 10.9	↑						↓ hs-CRP and platelet count; ↑ the quality of life; Did not alter levels of WBC and ESR
Papada et al. (2018) [27]	Remission DC and UC	Randomized double blind, placebo control	*Pistacia lentiscus*	2800 mg/d For 12 weeks	Placebo: n = 27; age = 45 y ± 17.4 Intervention: n = 33; age = 38.2 y ± 11.9			↑		Ox-LDL: ↓		↓ oxLDL/HDL, oxLDL/LDL and oxLDL/LDL
Karimi et al. (2019) [13]	Active mild to moderate UC	Randomized double blind	Vitamin D	Intervention 1: 1000 UI/d (I1) Intervention 2: 2000 UI (I2) For 12 weeks	I1: n = 22; age = 39.7 y ± 15.6 I2: n = 24; age = 34 y ± 12.5			NS				High dose group: ↑ the quality of life and ↓ severity of disease activity
Morshedzadeha et al. (2019) [14]	UC	Randomized double blind, placebo control	Intervention 1: Grounded flaxseed (GF) Intervention 2: Flaxseed oil (FO)	GF: 30,000 mg/d FO: 10,000 g/d For 2 weeks	Placebo: n = 25; age = 35.2 y ± 10.6 GF: n = 25; age = 29.9 y ± 9.1 FO: n = 25; age = 32.2 y ± 9.9						IL-6 and IFN-γ: GF and FO (↓)	GF and FO: ↑ TGF-β and the quality of life; ↓ fecal calprotectin, Mayo score, ESR, waist circumference, diastolic and systolic blood pressure
Nikkhah-Bodaghi et al. (2019) [15]	Active mild to moderate UC	Randomized double blind, placebo control	*Nigella sativa*	2000 mg/d For 6 weeks	Placebo: n = 24; age = 39.2 y ± 11.8; Intervention: n = 24; age = 34.8 y ± 11.2			NS	↓	NFκB: NS	TNF-α: NS	↓ stool frequency score; Did not alter severity of disease activity and the quality of life
Nikkhah-Bodaghi et al. (2019) [16]	Active mild to moderate UC	Randomized double blind, placebo control	Zingiber	2000 mg/d For 12 weeks	Placebo: n = 24; age = 39.2 y ± 11.8 Intervention: n = 22; age = 41.4 y ± 11.4			NS	↓			↓ severity of disease activity; ↑ the quality of life
Abhari et al. (2020) [17]	Active mild to moderate UC	Randomized double blind, placebo control	Omega 3	4300 mg/d For 8 weeks	Placebo: n = 35; age = 69.7 y ± 5.0. Intervention: n = 35; age = 69.7 y ±5.5	↑	↑		↓	Catalase: ↑ Ox- LDL: ↓	IL-6, IL-2, IL-1α and IL-1β: ↓	Did not alter BMI, waist circumference, diastolic and systolic blood pressure
von Martels et al. (2020) [29]	DC and UC	Prospective	Riboflavin	100 mg/d For 3 weeks	Group 1 (Fecal Calprotectin < 200 µg/g): n = 40; age = 44.2 y ± 11.6 Group 2 (Fecal Calprotectin > 200 µg/g): n = 30; age = 38.8 y ± 13.6					Free thiols: ↑	IL-6, IL-10, TNF-α and IL-1β: NS IL-2: ↓	↓ severity disease activity, CRP and Enterobacteriaceae; No effects on diversity, taxonomy, or metabolic pathways of the fecal microbiome.
Farsi et al. (2021) [18]	Varying disease activity UC	Randomized double blind, placebo control	Coenzyme Q10	200 mg/d For 8 weeks	Placebo: n = 43; age = 40.2 y ± 11.5 Intervention: n = 43; age = 38.4 y ± 8.8						IL-10: ↑ IL-17: ↓	↓ severity disease activity; ↑ the quality of life and serum levels of cathelicidin LL-37; Did not alter β-defensin 2
Tahvilian et al. (2021) [19]	Active mild to moderate UC	Randomized double blind, placebo control	Saffron	100 mg/d For 8 weeks	Placebo: n = 35; age = 41.0 y ± 11.3 Intervention: n = 40; age = 40.5 y ± 12.7	↑	↑	↑	NS			
Tavassolifar et al. (2021) [25]	Active mild to moderate CD	Longitudinal	Azatioprine	50 mg/d For 12 weeks	Healthy control: n = 15; age = 33.6 y ± 1.2 CD patients: n = 15; age = 31.5 y ± 1.8	Normalized *				GP91PHOX, NrF2, Catalase—normalized *		↓ severity disease activity
Khazdouz et al. (2023) [30]	Active mild to moderate UC	Randomized double blind, placebo control	Selenium	200 mcg/d 10 weeks	Placebo: n = 50; age = 37.9 ± 10.8 Intervention: n = 50; age = 34.5 ± 11.2						IL-17 ↓ IL-10 (NS)	↓ severity disease activity; ↑ the quality of life

Legend: * = gene expression; n = total number; ↑ = increased; ↓ = reduced. AOC: Antioxidant capacity; DC: Crohn’s disease; ESR—erythrocyte sedimentation rate; GP91PHOX: 91-kD glycoprotein component; GPx: Glutathione peroxidase; HDL: high density lipoprotein IFN-γ: Interferon gamma; IQ: interquartile range; IL: Interleukin; LDL: low density lipoprotein; LP: Lipid peroxidation; MDA: malondialdehyde; NFκB: nuclear factor kappa B; Nrf2: nuclear factor erythroid 2-related factor 2; NS: not significant; Ox-LDL: oxidized low-density lipoprotein; SEM: standard deviation of mean; SD: standard deviation; SOD: Superoxide dismutase; TGF-β: transforming growth factor beta; TNF- α: tumor necrosis factor alpha; UC: Ulcerative colitis; Vit: Vitamin; WBC: white blood cells y: Years; SEM: standard error of the mean.

**Table 2:** Bias risk of randomized included studies.

	DOM 1	DOM 2	DOM 3	DOM 4	DOM 5	DOM 6	Overall
Mulder, et al. (1994) [20]	Unclear	Unclear	Unclear	Unclear	Unclear	Unclear	Unclear
Geerling et al. (2000) [21]	Unclear	Unclear	Unclear	Unclear	Unclear	Unclear	Unclear
Barbosa et al. (2003) [11]	Unclear	Unclear	Unclear	Low	High	Unclear	Unclear
Aghdassi et al. (2003) [22]	Low	Unclear	Unclear	Unclear	High	High	Unclear
Akobeng et al. (2007) [23]	Low	Low	Low	Low	Low	High	Low
Samsamikor et al. (2016) [12]	Unclear	Unclear	Low	Low	Low	High	Low
Nematgorgani et al. (2017) [26]	Unclear	Low	Low	Low	Low	High	Low
Papada et al. (2018) [27]	Low	Low	Low	Low	Low	Low	Low
Ballini et al. (2019) [28]	Low	Low	Unclear	Unclear	Low	Low	Low
Nikkhah-Bodaghi et al. (2019) [15]	Low	Low	Low	Low	High	Unclear	Low
Nikkhah-Bodaghi et al. (2019) [16]	Low	Low	Low	Low	High	Unclear	Low
Karimi et al. (2019) [13]	Unclear	Low	Low	Unclear	Low	Unclear	Unclear
Morshedzadeh et al. (2019) [14]	Unclear	Unclear	High	Unclear	Low	Low	Unclear
Tahvilian et al. (2020) [19]	Low	Low	Low	Unclear	Low	Low	Low
Abhari et al. (2020) [17]	Unclear	Unclear	High	High	High	High	High
Farsi et al. (2021) [18]	Low	Low	Low	Low	Low	Low	Low
Khazdouz et al. (2023) [30]	Low	Low	Low	Low	Low	Low	Low

Legend: DOM 1: Sequence generation; DOM 2: Allocation concelament; DOM 3: Blinding of participants and professionals; DOM 4: Blinding of outcome assessors; DOM 5: Incomplete outcomes; DOM 6: Selective report.

**Table 3:** Bias risk of non-randomized included studies.

	DOM 1	DOM 2	DOM 3	DOM 4	DOM 5	DOM 6	DOM 7	Overall
von Martels et al. (2020) [29]	Moderate	Low	Low	Low	Moderate	Low	Low	Moderate
Koláček et al. (2013) [24]	Low	Low	Low	Low	Low	Low	Low	Low
Tavassolifar et al. (2021) [25]	Moderate	Low	Low	Low	Low	Low	Low	Low

Legend: DOM 1: Confounding; DOM 2: Selection of participants into this study; DOM 3: Classification of interventions; DOM 4: Deviations from intended interventions; DOM 5: Missing data; DOM 6: Measurement of outcomes; DOM 7: Selection of the reported result.

## Data Availability

Data sharing is not applicable.
